# Occupational Therapy Interventions Using New Technologies in Children and Adolescents with Autism Spectrum Disorder: A Scoping Review

**DOI:** 10.1007/s10803-022-05431-3

**Published:** 2022-01-20

**Authors:** S. Domínguez-Lucio, L. M. Compañ-Gabucio, L. Torres-Collado, M. García de la Hera

**Affiliations:** 1 Unidad de Epidemiología de la Nutrición, Departamento de Salud Pública, Historia de la Ciencia y Ginecología, Universidad Miguel Hernández (UMH), 03550 Alicante, Spain; 2 de Investigación Sanitaria y Biomédica de Alicante, ISABIAL-UMH, 03010 Alicante, Spain; 3 CIBER Epidemiología y Salud Pública (CIBERESP), Instituto de Salud Carlos III, 28034 Madrid, Spain

**Keywords:** Autism spectrum disorder, New technologies, Occupational therapy, Rehabilitation, Computer, iPad

## Abstract

New technologies (NT) are increasingly used in Occupational Therapy (OT) interventions in people with Autism Spectrum Disorder (ASD). We conducted a scoping review to describe OT interventions investigated in scientific literature which use NT in children and adolescents with ASD. Two authors independently searched the scientific databases PubMed, EMBASE, Scopus and Web of Science, carried out a peer-review screening of articles and extracted data. Twenty studies met the inclusion criteria. OT interventions lasted between 1 week and 12 months, consisted of between 1 session per day and 5 sessions per week, were carried out exclusively by an occupational therapist, and the most used NT was the computer (n = 12). The duration of the interventions and assessment measures used varied greatly between studies.

**Supplementary Information:**

The online version contains supplementary material available at 10.1007/s10803-022-05431-3.

## Introduction

Autism Spectrum Disorder (ASD) is a neurodevelopmental disorder characterized by difficulties in social communication across multiple contexts and by unusually restricted and repetitive behaviors, interests or activities [American Psychiatric Association (APA), 2013; Lai et al., 2014]. The worldwide prevalence of ASD varies between 1 and 2% of the population (Elsabbagh et al., 2012), but it is considerably higher in the United States and South Korea [World Health Organization (WHO), 2021]. In Spain, ASD prevalence has increased over the last 40 years and differs greatly between studies, showing a prevalence of between 0.02 and 1.5% (Málaga et al., 2019).

ASD has a direct impact on peoples’ occupation which limits their autonomous and independent functioning (Lamash & Josman, 2020; Weaver, 2015), especially among children and adolescents (Huang et al., 2020; Zhang, 2002). Children and adolescents with ASD have difficulty initiating and maintaining interactions with their peers, such as playing or social activities (Gal et al., 2016; Taylor et al., 2017). Play is the most important occupation for children because it contributes to all areas of child development and facilitates the acquisition of the performance skills required for activities of daily living. In addition, in adolescents, play is also a way to improve different ASD-behaviors, family relationships as well as socialization and communication skills (Lindsay et al., 2017). Play absence or impairment can, therefore, impact negatively on their occupational performance in adult life (Blázquez-Ballesteros et al., 2015). Thus, early detection of ASD and early interventions in this population are essential to reduce their limitations in daily life (Sánchez-Raya et al., 2015). In this early intervention, the occupational therapist assumes an important role as they intervene directly on the autonomy and independence of children and adolescents with ASD (Lamash & Josman, 2020; Weaver, 2015) in different settings such as home and school (Tanner et al., 2015; Tomchek et al., 2017). These professionals use occupation and meaningful activity, in this population can be playing, in a structured way as a means of rehabilitation (Muñoz & Noriega, 2016) and they develop the intervention taking into account the preferences, needs and abilities of people with ASD (Weaver, 2015).

A systematic review published in 2017 (Hollis et al., 2017) described a rapid growth and evolution of assessments and interventions using new technologies (NT) in children and adolescents with ASD, carried out by different health professionals, including occupational therapists (Hollis et al., 2017; Odom et al., 2015). NT are defined as the study and application of current digital technologies and telecommunication systems (Lorido, 2005). In particular, new technologies for rehabilitation are ‘‘newly developed mechanical or computer systems, often involving microprocessors or computer hardware and software’’ used by a therapist ‘‘to remediate impairment, promote recovery, and improve peoples’ function’’ (Chen & Bode, 2011). Intervention studies using NT have used a variety of devices such as computers, smartphones, tablets, robots, virtual reality and wearable technology, with the aim of increasing communication, social, emotional and academic skills in children and adolescents with ASD (Sandgreen et al., 2020). Specifically, scientific evidence on Occupational Therapy (OT) intervention using NT in children and adolescents with ASD has shown beneficial effects in children’s academic skills (literacy and numeracy) (Shic & Goodwin, 2015), the performance of activities of daily living (Becker et al., 2016; Shic & Goodwin, 2015) as well as in the acquisition of social skills (interaction and collaboration with their peers) (Bauminger-Zviely et al., 2013; Shic & Goodwin, 2015).

Despite the fact that the results obtained in different studies suggest a positive effect of NT for children and adolescents with ASD (Lamash & Josman, 2021; Odom et al., 2015; Sandgreen et al., 2020; Shic & Goodwin, 2015), some studies pointed out that scientific evidence on the efficacy of these interventions is still inconclusive (Lorido, 2005; Odom et al., 2015). However, NT improve access to interventions because they can be performed remotely, and therefore allow more adaptive, interactive and predictable interventions (Hollis et al., 2017). In addition, NT are a motivating and attractive intervention for children and adolescents because today, children and adolescents belong to the "digital generation" for whom technology forms part of daily life from a very early age (Odom et al., 2015). For these reasons, NT are a very useful tool to complement OT intervention for children and adolescents with ASD. Based on this background, two research questions emerged: (1) What are the most commonly used NT in OT interventions in children and adolescents with ASD in the scientific literature? (2) What are the characteristics of OT interventions with NT in children and adolescents with ASD investigated in the scientific literature? The aim of this scoping review was to describe OT interventions investigated in scientific literature which use NT in children and adolescents with ASD.

## Methods

A scoping review was conducted following the standards of the Cochrane Handbooks Version 6.2, 2021 (Higgins & Thomas, 2021) and the recommendations of the PRISMA Extension for Scoping Reviews (PRISMA-ScR) (Tricco et al., 2018). We have not published a protocol of this review nor registered it on PROSPERO or any other similar scientific website.

### Search Strategy

We performed a systematic literature search on the databases MEDLINE (PubMed), EMBASE, Scopus and Web of Science (WOS) on November 16, 2020. We performed an additional manual search of several OT journals indexed in the Journal Citation Reports (JCR) including those with the highest impact (indexed in the first quartile): American Journal of Occupational Therapy (AJOT); Australian Occupational Therapy Journal (AOJT); British Journal of Occupational Therapy (BJOT); Canadian Journal of Occupational Therapy (CJOT); Hong Kong Journal of Occupational Therapy (HKJOT); Journal of Occupational Rehabilitation (JOR); Occupational Therapy International (OTI); Occupation, Participation and Health (OTJR); Physical and Occupational Therapy in Pediatrics (POTP); Scandinavian Journal of Occupational Therapy (SJOT). We used the same search strategy in all the sources consulted, including all ASD disorders, ‘occupational therapy’, and different terms included in NT as search terms, combined with the Boolean operators AND and OR. Search strategies used in all databases can be found in Table 1.

**Table 1:** Databases and search strategies used

Databases	Search strategy 16-11-2020
PUBMED	
#1	(ASD OR autism OR autistic OR asperger OR Rett OR pervasive OR disintegrative)
#2	"occupational therapy"
#3	(app OR apps OR application) OR (tablet OR ipad) OR mobile OR “virtual reality” OR (computer OR laptop) OR technology OR device OR internet OR ("video game" OR software)
	#1 AND #2 AND #3
SCOPUS	
	ALL ( ( asd OR autism OR autistic OR asperger OR rett OR pervasive OR disintegrative))
	ALL ( "occupational therapy")
	ALL ( ( app OR apps OR application) OR ( tablet OR ipad) OR mobile OR "virtual reality" OR ( computer OR laptop) OR technology OR device OR internet OR ( "video game" OR software))
	( TITLE-ABS-KEY ( asd OR autism OR autistic OR asperger OR rett OR pervasive OR disintegrative) AND ALL ( "occupational therapy") AND ALL ( app OR apps OR application OR tablet OR ipad OR mobile OR "virtual reality" OR computer OR laptop OR technology OR device OR internet OR "video game" OR software))
Web of science	
	((ASD OR autism OR autistic OR asperger OR rett OR pervasive OR disintegrative))
	("occupational therapy")
	((app OR apps OR application) OR (tablet OR ipad) OR mobile OR “virtual reality” OR (computer OR laptop) OR technology OR device OR internet OR ("video game" OR software))
	((ASD OR autism OR autistic OR asperger OR rett OR pervasive OR disintegrative)) AND #2 ("occupational therapy") AND #3 ((app OR apps OR application) OR (tablet OR ipad) OR mobile OR “virtual reality” OR (computer OR laptop) OR technology OR device OR internet OR ("video game" OR software))
EMBASE	
#1	'asd'/exp OR asd OR 'autism'/exp OR autism OR autistic OR asperger OR rett OR pervasive OR disintegrative
#2	'occupational therapy'
#3	app OR apps OR application OR tablet OR ipad OR mobile OR 'virtual reality' OR computer OR laptop OR technology OR device OR internet OR 'video game' OR software
	#1 AND #2 AND #3

### Review Criteria

In this review, we included all articles that met the following inclusion criteria: (a) Studies with experimental design: non-randomized controlled trials, randomized controlled trials, quasi-experimental studies, pilot studies, case report and exploratory studies; these types of studies are included in the levels of evidence 2,3,4 established by the Oxford Centre for Evidence-Based Medicine (Centre for Evidence-Based Medicine (CEBM) n.d.); (b) children or adolescent population (under 18 years old); (c) study population with a diagnosis of ASD: Asperger's Syndrome, Rett Syndrome, Disintegrative Disorder, Classic Autistic Disorder, and Pervasive Developmental Disorder, (d) OT intervention using NT: app, tablet, iPad, mobile, virtual reality, computer, laptop, technology device, internet, video game or software. We excluded the following: (a) studies not published in English and/or Spanish; and (b) with no full-text available.

No filters by time or type of study were applied during the literature search in any of the databases and journals consulted.

### Study Selection

We downloaded and compiled all the article titles obtained from all the searches using Microsoft Excel for further review and screening. One researcher (SDL) carried out a preliminary screening which consisted of removing duplicates. Two researchers (SDL and LMCG) then reviewed and screened the articles independently. We performed three comprehensive, successive and screenings: by title, by abstract and by full text. In each screening, articles that did not meet the inclusion criteria were discarded. A third review research (MGDH) resolved discrepancies between SDL and LMCG regarding study inclusion at the end of each of the three screenings.

### Data Extraction and Synthesis

Before the data extraction, all researchers designed the tables and defined the items to be included in them, in order to reduce subjectivity in the synthesis of the data*.* We elaborated three tables based on the Cochrane Handbook (Higgins & Thomas, 2021). Table 2, which provides information on the main characteristics of the studies included in this review through the following items: author/year, study design, sample/country, participants, intervention/comparator, evaluation, and study outcomes (Page et al., 2021). Table 3, which provides information on the main characteristics of the interventions conducted in the studies included in this review through the following items: author/year, participants, intervention, duration of intervention, sessions, intervention manager and main results (Page et al., 2021). And finally, Table 4, which provides information regarding aspects related to the risk of bias in the articles included in this review through the following items: author/year, main limitations, funding sources and declarations of interest (Boutron et al., 2021). Two researchers (SDL and LMCG) performed the data extraction independently, LTC resolved discrepancies about data extraction.

**Table 2:** Main characteristics of the included studies

Author, year	Study design	Sample, country	Participants	Intervention/comparator	Evaluation	Study outcomes
Josman et al. (2008)	Pilot	12, Israel Loss of follow-up n = 0	IG: 3 boys and 1 girl with ASD CG: 5 boys and 1 girl with TD Age: 8–16 years	Street-crossing desktop virtual environment/ Street-crossing desktop virtual environment	Pre- and post- evaluation	Pedestrian behavior and safety when crossing the street assessed with the PSS
Cosper et al. (2009)	Quasi	12, United States Loss of follow-up n = 0	10 boys and 2 girls with ASD and ADHD or CDD Age: 6–13 years	Computer-based noninvasive technology (Interactive Metronome)/NA	Pre- and post- evaluation	Motor control and coordination assessed with the *BOT-2 Brief* Sustained attention assessed with the *GDS*
Lo et al. (2009)	Pilot	3, Taiwan Loss of follow-up n = 0	3 boys with ASD and 1 UND Age: 4–7 years	Interactive game: Racing Game Playful Tray and Interactive game: Playful Toothbrush/NA	Pre- and post- evaluation	Duration of meals and mealtime behavior assessed with video recordings Efficiency and number of brushings assessed with a plaque-revealing dye and video recordings
Gentry et al. (2010)	Quasi	22, United States Loss of follow-up n = 0	18 boys and 4 girls with ASD Age: 14–18 years	Training intervention in the use of a PDA as a task management tool/NA	Pre- and post-evaluation	Occupational performance assessed with the *COPM* PDA use assessed with the *FATCAT*
Wuang et al. (2010)	Quasi	60, Taiwan Loss of follow-up n = 11	47 boys and 13 girls with ASD Age: 6–10 years	Simulated Developmental Horse-Riding Program + OT/OT	Pre-, 22-week and post- evaluation	Motor function assessed with the *BOT-2* Sensory integration assessed with the *TSIF*
Palsbo and Hood-Szivek (2012)	Pilot	18, United States Loss of follow-up n = 2	14 boys and 4 girls with ASD, ADHD, PDD, ID, APD or UN Age: 5–11 years	Robotic-guided three-dimensional repetitive fine motion training/NA	Pre- and post- evaluation	Motor control assessed with the *VMI* Handwriting skills assessed with the *THS–R, the Print Tool and the ETCH*
Lee et al. (2013)	nRCT	2, United States Loss of follow-up n = 0	2 boys with ASD Age: 4–5 years	Computer-based intervention program for enhancing visual perceptual skills/NA	Pre- and post-evaluation	Visual perception assessed with the *DTVP-2*
Janeslätt et al. (2014)	RCT	47, Sweden Loss of follow-up n = 10	25 boys and 12 girls with ADHD, ASD, CP, ID or SB Age: 6–11 years	Compensatory intervention based on the use of time management aids/ Usual therapy + Compensatory intervention based on the use of time management aids	Pre- and post-evaluation	Time management skills assessed with the *KaTid-Child* and the *Time-Parent scale*
Lorah et al. (2014)	Case	3, United States Loss of follow-up n = 0	2 boys and 1 girl with ASD and CH Age: 4–6 years	Use of the iPad™ and application Proloqu2Go as a speech generating device/NA	Pre- and post-evaluation	Speech generation and sentence discrimination assessed with the Proloquo2Go trial scores
Campbell et al. (2015)	nRCT	3, United States Loss of follow-up n = 0	2 boys and 1 girl with ASD Age: 17–19 years	Video modeling on a handheld device to teach hand washing (Sylvania HD Video Mp4 Player)/NA	Pre- and post-evaluation	Occupational performance assessed with a video task analysis
Chen et al. (2015)	Case	3, Taiwan Loss of follow-up n = 0	3 adolescents with ASD (sex NS) Age:10–13 years	Augmented reality technology/NA	Pre- and post- evaluation	Emotion recognition assessed with video stories questions
Meister and Salls (2015)	Pilot	9, United States Loss of follow-up n = 0	8 boys y 1 girl with ASD Age: 7.5–13.5 years	Point-of-view video modeling as an intervention strategy to improve self-help skills using iPad/NA	Pre- and post- evaluation	ADLs assessed with the *COSA*
Gal et al. (2016)	Quasi	14, Israel Loss of follow-up n = 0	14 boys with ASD Age: 8–12 years	StoryTable application implemented through the Diamond Touch multitouch tabletop/NA	Pre- and post- evaluation and 3-week follow up	Social interaction assessed with the *FOS*
Henning et al. (2016)	Pilot	10, Australia Loss of follow-up n = 0	5 boys with ASD and 3 boys and 2 girls with TD Age: 4–11 years	Play-based intervention, including therapists, peers, parents and video modeling/NA	Pre- and post- evaluation and 2-month follow-up	Play skills assessed with *the ToP* Social and behavioral problems assessed with the *CCBRS-P*
Ikuta et al. (2016)	Pilot	21, Japan Loss of follow-up n = 0	16 boys and 5 girls with ASD Age: 4–16 years	Control period followed by Earmuffs and finally Headphones/Control period followed by Headphones and finally Earmuffs	Pre- and post- evaluation	Effectiveness of the intervention assessed with the *GAS*
Lee et al. (2016)	Quasi	6, Taiwan Loss of follow-up n = 0	4 boys and 2 girls with ASD Age: 12–15 years	Applied Cliplets‑based half‑dynamic videos as intervention/NA	Pre- and post- evaluation and 1-month follow-up	Emotion recognition assessed with the analysis of facial expressions on video
Hatfield et al. (2017)	nRCT	94, Australia Loss of follow-up n = 6	IG: 39 boys and 10 girls with ASD CG: 33 boys and 12 girls with ASD Age: 12–18 years	Better Outcomes & Successful Transitions for Autism (BOOST-A™): online/NA transition planning program/Regular transition planning practice	Pre- and post- evaluation	Self-Determination assesses with the *AIR*
Hochhauser et al. (2018)	RCT	61, Israel Loss of follow-up n = 10	IG: 32 boys and 4 girls with ASD CG: 2 girls and 23 boys with ASD Age: 12–18 years	Computer application that uses video modeling and video self-modeling: CONTACT/No intervention	Pre- and post- evaluation and 1-month follow-up	Negotiation attitudes and behaviors assessed with the *FFNS* Conflict management assessed with *The ConflicTalk questionnaire*
Lamash and Josman (2021)	nRCT	56, Israel Loss of follow-up n = 0	IG: 29 boys and 4 girls with ASD CG: 17 boys and 6 girls with ASD Age: 11–19 years	Virtual supermarket (VAPs) + metacognitive intervention/Standard intervention to promote shopping	Pre- and post- evaluation	Cognitive functions assessed with the *WebNeuro® software* Shopping in the community assessed with the *TOGSS*
Parsons et al. (2019)	RCT	59, Australia Loss of follow-up n = 12	IG: 25 boys and 5 girls with ASD CG: 23 boys and 6 girls with ASD Age: 2–6 years	iPad application: The Therapeutic Outcomes by You application (TOBY app)/Usual therapy and iPad without the TOBY app installed	Pre- and post- evaluation and 6-month follow-up	Cognitive function assessed with the *MSEL* Social imitation assessed with the *CSBS*

*ADHD* attention deficit hyperactivity disorder, *AIR* Self-Determination Scale, *APD* auditory processing disorder, *ASD* autism spectrum disorder, *BOT-2* brief Bruininks–Oseretsky test of motor proficiency-short version, *Case* case series, *CCBRS-P* Conners’ Comprehensive Behaviour Rating Scales–parent, *CDD* developmental coordination disorder, *CG* control group, *CH* cerebellar hypoplasia, *COPM* Canadian occupational performance measure, *COSA* child occupational self-assessment, *CP* cerebral palsy, *CSBS* Communication and Symbolic Behavior Scales*, DTVP-2* developmental test of visual-perceptual skills-2, *ETCH* evaluation tool of children’s handwriting, *FATCAT* functional assessment tool for cognitive assistive technology, *FFNS* Five Factor Negotiation Scale, *FOS* Friendship Observation Scale, GAS Goal Attainment Scaling, *GDS* gordon diagnostic system, *ID* intellectual disability, *IG* intervention group, *KaTid-Child* Kit for assessing time-processing ability in children, *MSEL* Mullen Scales of early learning, NA not applicable, *nRCT* non-randomized controlled trial, *NS* not stated, *PDA* personal digital assistant, *PDD* pervasive developmental disorder, *PSS* Pedestrian Safety Scale, *Pilot* pilot study, *Quasi* quasi-experimental study, *RCT* randomized controlled trial, *SB* spina bifida, *TD* typical development, *THS–R* test of handwriting skills–revised, *TOGSS* test of grocery shopping skills, *ToP* test of playfulness, *TSIF* test of sensory integration function, UN undiagnosed*, VMI* Beery–Buktenica developmental test of visual–motor integration

**Table 3:** Characteristics of the interventions performed in the included studies

Author, year	Participants and diagnosis	NT	Interventions	Duration (w)	Sessions	Intervention managers	Main results
Josman et al. (2008)	12, ASD and TD	Computer	IG: Street-crossing desktop virtual environment (VE). 9 stages. The VE was designed as a typical four-lane divided street, had two types of cross walks, one with a pedestrian island and one with a traffic light CG: Same as IG	NS	IG: One- to two-weekly 10-to 30-min sessions CG: one 45-min session	OTs	Low number of sessions to successfully complete the 9 stages needed in IG vs CG (p < 0.01) Higher task performance in post- vs pre- IG scores (p < 0.01)
Cosper et al. (2009)	12, ADHD, CDD and ASD	Computer	Interactive Metronome. Non-invasive PC-based technique to practice the timing and rhythmicity of various movement combinations of the hands and feet in response to auditory cues	15	One-weekly 1-h sessions	OTs	Increase in complex visual choice reaction time (p < 0.05), in visuomotor control (p = 0.02), in UL balance and coordination (p = 0.06) and in UL speed (p = 0.07), in post-vs pre- intervention
Lo et al. (2009)	4, ASD and UN	Computer	Racing Game tray. Interactive game over a weight-sensitive tray surface. The tray surface recognizes and follows the child's natural eating actions in real time. When the child completes the meal, the game ends. Playful Toothbrush. Interactive game into a brushing activity. The game starts with a virtual image of uncleaned teeth, when the child finishes cleaning all his or her teeth, the virtual teeth become completely white and an applause sound plays	4	One-daily session. Duration NS	OTs	33% reduction in meal duration and 20% reduction in non-food related behaviors in post-vs pre- intervention Increase in cleaning effect (from 32 to 67%.) and in the average number of brushing strokes (from 190 to 248) in post-vs pre- intervention
Gentry et al. (2010)	22, ASD	Another NT (PDA)	Software Palm® Desktop was used onto the computer where calendar entries and alarms are entered and transferred to the PDA via USB. They added medication schedules, homework, household chores and other calendar items to the PDA, adding a reminder alarm to each	6	One-weekly 60- to 90- min sessions	OTs	Increase in performance and satisfaction with performance of everyday life tasks (p < 0.001) in post-vs pre- intervention
Wuang et al. (2010)	60, ASD	Another NT (Simulated program)	Simulated Developmental Horse-Riding Program. Joba® is an exercise equipment that simulates the movements during real horseback riding, for the improvement of motor and sensory integration functions. A intervention plan was especially designed for each child that incorporated activities compatible with the child’s interest and current motor function	40	Two-weekly 1-h sessions	OTs	Increase in motor function (p < 0.0001) and sensory integration (p < 0.0001) in post- vs pre- intervention
Palsbo and Hood-Szivek (2012)	18, ASD, ADHD, PDD, ID, APD, CP and UN	Another NT (Robot)	Robotic-guided three-dimensional repetitive fine motion training to improve handwriting. *My Scrivener* haptic interface device. During sessions 10-min review of the letters and numbers, 10 min with robot-assisted glyph formation and 10 min on the workbook lesson were offered	4–6	Three- to five-weekly 30-min sessions	OTs and research professor	Improvement in motor control percentiles (from 29 to 42, p < 0.10) in post- vs pre- intervention Improvement in consistency of glyph formation in 10 children (p < 0.10) in post- vs pre- intervention
Lee et al. (2013)	2, ASD	Computer	*Concepts on the Move-Basic* and *Concepts on the Move 2-Advanced* software. to improve visual-perceptual skills. The participants chose three of the nine items for each of the five categories, such as colors, sizes or shapes, yielding 15 permutations per session	6	Two-weekly 30-min sessions	OTs	Improvement in motor-reduced visual-perceptual skills in post- vs pre- intervention. (p value NS)
Janeslätt et al. (2014)	47, ADDH, ASD, CP, ID and SB	Another NT (PDA)	IG: Received at least one-time aids for daily time management, based on devices that make the time passing visible CG: Received intervention as usual during their waiting-list time. During intervention they received the same intervention as IG	NS	Two- to five weekly 1- to 2-h sessions	OTs and special educator	Increase in time processing ability (p < 0.05).in IG vs CG
Lorah et al. (2014)	3, ASD	iPad™	Proloqu2Go app as a speech generating device for the acquisition of a tact (labeling) repertoire. A small plastic toy dog, a small ball, a standard size crayon and a children's book were used as stimuli during training	NS	One-daily sessions. Duration NS	OTs	Improvement in the acquisition of a tact repertoire and in the discrimination between the acquired sentence frames (p value NS)
Campbell et al. (2015)	3, ASD and PDD	Computer	Video modeling intervention using a handheld device (Sylvania HD Video Mp4 Player) to teach hand washing. The video was elaborated by 13-step task analysis	4	NS	OTs	Increased levels of handwashing skill and independence in post- vs pre- intervention (p value NS)
Chen et al. (2015)	3, ASD	Computer	Training emotional judgments and social skills through 3D Augmented Reality Self-Facial Modeling system. Participants read the scenario script and looked at the corresponding illustrations on the monitor screen to select one of the six basic emotional masks to wear	6	One-weekly 1-h sessions	OTs	Improvement in emotional and expression recognition in post- vs pre- intervention (p < 0.05)
Meister and Salls (2015)	8, ASD	iPad™	Video intervention to improve self-help skills. A video was created demonstrating each step of the target activity, and included simple step-by-step verbal directions. The *iMovie* and *VideoTote* applications were used	6	Two-weekly 10-to 25- min sessions	OTs	Improvement of 50.5% in post- vs pre- intervention (p value NS)
Gal et al. (2016)	14, ASD	Computer	*StoryTable *(*ST*) application. Intervention based on a collaborative narrative tabletop interface to improve social skills*.* Children were teaching in different aspect of social interaction, such as sharing, negotiating and receiving and providing help. Then, children were asked to narrate a joint story using the ST functions	3	Three- to four- 45-min sessions	OTs	Increase in positive social interactions (p = 0.004) and collaborative play (p = 0.007) in post- vs pre- intervention Decrease in negative social interaction (p = 0.05) in post- vs pre- intervention
Henning et al. (2016)	10 ASD and TD	Computer	Play-based intervention to improve social play interactions. Video feedback and play in the clinic which consisted of watching an episode of the DVD, discussing what they had seen and arranging a weekly play date with the playmate	7	9-weekly sessions. Duration NS	OTs	Improvements in the mean ToP score from 39.8 to 54.1 in post- vs pre- intervention
Ikuta et al. (2016)	21, ASD	Another NT (Earmuffs and Noise-cancelled headphones)	Intervention through standard earmuffs and noise-cancelled headphones in controlling behavioral problems related to hyper-reactivity to auditory stimuli	6	NS	OTs	Higher effectiveness of intervention score for the earmuff period than that for the control period (p = 0.006) There were no significant differences between the noise-cancelled headphone period and the control period (p = 0.91), and between the earmuff period and noise-cancelled headphone period (p = 0.67)
Lee et al. (2016)	6, ASD	Computer	Intervention using Applied Cliplets‑based half‑dynamic videos. Children had to selected the basic facial expression that they thought best reflected the feelings of the character in the video and one of six adjectives to answer each question, and received corrective feedback from the therapist	6	One-weekly 1-h sessions	OTs	High correct assessment rates of all the participants in post- vs pre- intervention (p < 0.05)
Hatfield et al. (2017)	94, ASD	Computer	IG: Autism specific online program *The Better OutcOmes & Successful Transitions for Autism*. This program consisted of four modules delivered via a website that is accessed by an individual login CG: Regular practice at their respective schools. This may have included any generic transition planning processes utilized at the school	48	NS	OTs	There were no significant differences between IG and CG, in self-determination according to AIR comparing pre- and post-intervention for adolescents (p = 0.19) Improvement in career exploration (p = 0.01) in IG vs CG
Hochhauser et al. (2018)	61, ASD	Computer	IG: CONTACT, computer application that uses video modeling and video self-modeling to address conflict resolution skills CG: no intervention	6	One-weekly 60-min sessions	OTs and researchers	Improvements in self-confidence (p = 0.02), communication (p = 0.003) and negotiation skills (p ≤ 0.001) in IG vs CG
Lamash and Josman (2021)	56, ASD	Computer	IG: Metacognitive intervention combined with practice in a virtual supermarket. Virtual Action Planning-Supermarket (VAP-S) software was used. VAP-S simulates a regular supermarket, including selecting products according to a list that appears on the computer monitor, paying and exiting the supermarket CG: Standard OT intervention to promote a shopping task	8	8-weekly 45-min sessions	OTs	Improvements in the duration of the shopping task performance (p < 0.05), redundant entrances (p < 0.05), accuracy (p < 0.001) and total strategy use (p < 0.05) in IG vs CG
Parsons et al. (2019)	59, ASD	iPad™	IG: Therapy Outcomes by You (TOBY) iPad app, with four major skills areas: (1) visual motor; (2) imitation; (3) language; and (4) social CG: Usual therapy and iPad without the TOBY app installed	12	One-daily 20-min sessions	OTs and pshycologists	Increase in the sub-scale of receptive language (p = 0.039) in the MSEL, social (p < 0.001) and symbolic subdomains (p = 0.001) of the CSBS, in post- vs pre- intervention

*ADHD* attention deficit hyperactivity disorder, *APD* auditory processing disorder, *ASD* autism spectrum disorder, *CDD* developmental coordination disorder, *CG* control group, *CP* cerebral palsy, *CSBS* Communication and Symbolic Behavior Scales, *ID* intellectual disability, *IG* intervention group, *MSEL* Mullen Scales of early learning, NA not applicable, *NT* new technology, *NS* not stated, *OTs* occupational therapists, *PDA* personal digital assistant, *PDD* pervasive developmental disorder, *SB* spina bifida, *TD* typical development, *ToP* test of playfulness, *UL* upper limb, UN undiagnosed, *w* weeks

**Table 4:** Items related to risk of bias of included studies

Author, year	Main limitations	Funding sources	Declarations of interest
Josman et al. (2008)	NS	NS	NS
Cosper et al. (2009)	Lack of control group, small sample size, patients with comorbidity diagnosis, very little research to support Interactive Metronome training	NS	NS
Lo et al. (2009)	NS	NS	NS
Gentry et al. (2010)	Non-random, non-representative sample, small sample size, lack of control group, evaluation and training administered by the same people	Program development grant from the Commonwealth Neurotrauma Initiative Fund	NS
Wuang et al. (2010)	Restricted age range, lack of control group	NS	NS
Palsbo and Hood-Szivek (2012)	Absence of a valid instrument for functional measurement of fine motor control, small sample size, unable to assess the representativeness of all children with dysgraphia, improvements in self-esteem or social and family inclusion were not assessed	National Institutes for Disability Rehabilitation and Research Grant No. H133S070082 and by Obslap Research, LLC	NS
Lee et al. (2013)	Minimum number of participants (n = 2), results difficult to generalize, short intervention duration, high initial scores in the recognition subsection, which limited the monitoring of participants' progress	NS	NS
Janeslätt et al. (2014)	High dropout rate (21%), small sample size, high participants variability, measurement instruments have not been used previously to assess change over time	Clas Groschinskys Minnesfond and Centre for Clinical Research in Dalarna and Stiftelsen Sunnerdahls Handikappfond	The authors declare that they have no conflicts of interest
Lorah et al. (2014)	Highly structured and systematic nature of intervention procedures, generalization outside the training environment was not assessed, use of the same stimuli in all training sessions	NS	NS
Campbell et al. (2015)	Short study duration, small sample size	Did not receive funding support for the research, authorship and/or publication of this article	The authors declare that they have no conflicts of interest
Chen et al. (2015)	NS	NS	NS
Meister and Salls (2015)	Short study duration, consistency of performance over time not assessed, generalization in other contexts and at other times not assessed, lack of control group	NS	NS
Gal et al. (2016)	Small sample size, lack of control group	Autism speaks and the FBK-Haifa agreement	The authors declare that they have no conflicts of interest
Henning et al. (2016)	Small sample size, lack of randomization, methodological problems during home visits	NS	NS
Ikuta et al. (2016)	Small sample size, lack of control group, 4 participants refused to wear earmuffs or NC headphones, and 5 others refused to wear NC headphones (limited NC headphone period behavioral data), adverse and long-term effects were not examined, age, gender, general intelligence, functional level of participants, frequency of intervention, and duration of device use were not controlled	Grant-in-aid for Scientific Research (C) no. 21500473 from the Japan Society for the Promotion of Science	The authors declare that they have no conflicts of interest
Lee et al. (2016)	Small sample size, limited time for testing	Ministry of Science and Technology of Taiwan (MOST 104-2420-H-006-020-MY3)	The authors declare that they have no conflicts of interest
Hatfield et al. (2017)	The diagnosis of ASD was based on parental report and confirmed by SRS-2, high dropout rate for adolescents (10% control; 31% intervention), Quasi-random, non-blinded intervention assignment, no follow-up or information on work performance was collected	Australian Postgraduate Award scholarship from the Australian Federal Government and Curtin University. Financial support of the Cooperative Research Centre for Living with Autism (Autism CRC) under the Australian Government’s Cooperative Research Centers Program	The authors declare that they have read Biomed Central’s guidance on competing interests and wish to declare the following interests: MH developed the BOOST-A and was also the first author of the manuscript which describes the effectiveness of the BOOST-A
Hochhauser et al. (2018)	Measurement instrument with a low internal consistency, the transference of acquired behaviors to daily life was not assessed	The Erasmus Mundus Action 2 program of the European Union	NS
Lamash and Josman (2021)	Small sample size, ASD symptomatology was not directly measured, no follow-up, not designed as a randomized controlled trial, did not follow all CONSORT guidelines	NS	The authors declare that they have no conflicts of interest
Parsons et al. (2019)	Dosage and fidelity of intervention were lower than prescribed, high dropout rate (n = 12)	Lishman Health Foundation and support of South West Autism Group (SWAN) and Telethon Kids Institute	The authors (and funders) have no affiliation with the application or its developers and will not receive, or have previously received, any royalties from sales

*NS* not stated

We made a descriptive synthesis of the results and used tables and figures as far as possible to present the flow of the study selection process and the characteristics of the included studies. In addition, as a multidisciplinary research team, we discussed categories to classify the different types of OT interventions using NT in children and/or adolescents with ASD used in the included studies in the results section.

### Quality Assessment

The quality of the included studies was not assessed as this is not a mandatory requirement of scoping reviews (Arksey & O’Malley, 2005; Levac et al., 2010; Peters et al., 2020; Tricco et al., 2016, 2018). However, we have included a table about risk bias, as recommended in the Cochrane Handbook (Higgins & Thomas, 2021), which includes information on the main limitations reported in the included articles, funding sources and declarations of interest (Table 4) to make readers aware of these characteristics and to enable them to assess the results presented in this scoping review more critically. The main limitations reported by the authors of each included study are also discussed in the results section.

## Results

**Fig. 1:**
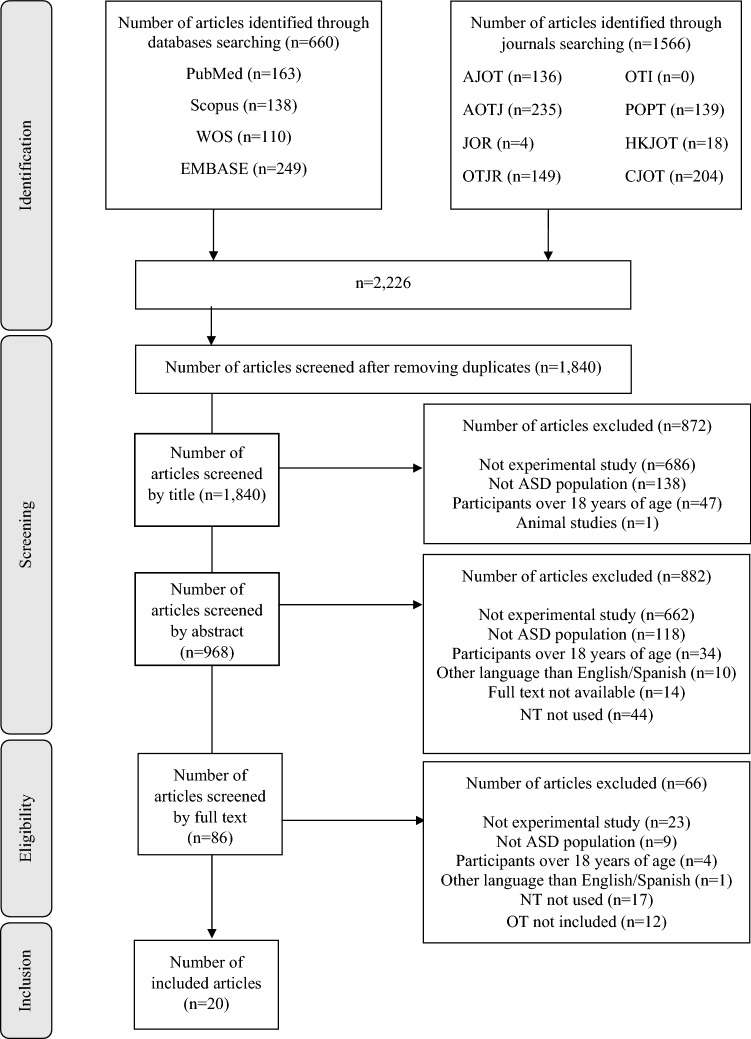
Flowchart of the study selection process

We identified 2226 published articles on OT intervention with NT in children and adolescents, which resulted in 1840 after removing duplicates. 1840 articles were screened by title, 968 by abstract and 86 by full text. Of these 86 articles, 20 met the inclusion criteria and were included in this scoping review. The study selection flowchart is shown in Fig. 1.

### Participants and sample

The main characteristics of the included studies are summarized in Table 2. Nine studies were conducted in Asian countries, seven in the United States, three in Australia and one in Europe. The study population of all the included articles consisted of children or adolescents with a diagnosis of ASD but nine of them, (Campbell et al., 2015; Cosper et al., 2009; Gal et al., 2016; Henning et al., 2016; Janeslätt et al., 2014; Josman et al., 2008; Lo et al., 2009; Lorah et al., 2014; Palsbo & Hood-Szivek, 2012) in addition to children and adolescents diagnosed with ASD, also included other diagnoses such as Attention Deficit Hyperactivity Disorder (ADHD), Intellectual Disability, Infantile Cerebral Palsy, Spina Bifida, Cerebellar Hypoplasia, Auditory Processing Disorder and undiagnosed population. The sample size varied greatly between included studies, two being the lowest number of participants (Lee et al., 2013), and ninety-four the highest (Hatfield et al., 2017).

### Study Design

Six of the included articles are pilot studies (two of them were randomized controlled clinical trials and four of them were quasi-experimental studies), (Henning et al., 2016; Ikuta et al., 2016; Josman et al., 2008; Lo et al., 2009; Meister & Salls, 2015; Palsbo & Hood-Szivek, 2012), five are quasi-experimental studies (Cosper et al., 2009; Gal et al., 2016; Gentry et al., 2010; Lee et al., 2016; Wuang et al., 2010), four are non-randomized clinical trials (Campbell et al., 2015; Hatfield et al., 2017; Lamash & Josman, 2021; Lee et al., 2013), three are randomized controlled clinical trials (Hochhauser et al., 2018; Janeslätt et al., 2014; Parsons et al., 2019), and two are case reports (Chen et al., 2015; Lorah et al., 2014). Only six of all included studies included a control group (Hatfield et al., 2017; Hochhauser et al., 2018; Janeslätt et al., 2014; Josman et al., 2008; Lamash & Josman, 2021; Parsons et al., 2019). In four of them (Hatfield et al., 2017; Hochhauser et al., 2018; Lamash & Josman, 2021; Parsons et al., 2019) the control group received either the usual therapy or received no intervention. In one study (Josman et al., 2008) both groups received the same intervention, the only difference being the number of sessions. Finally, in one study (Janeslätt et al., 2014) both groups received the same intervention, although the intervention group was treated before the control group, while the control group received a usual therapy. Two studies (Ikuta et al., 2016; Wuang et al., 2010) had two intervention groups, both of whom received the same intervention, although in different phases of the study or in a different order (Table 2).

### Study Variables and Measuring Instruments

Activities of daily living (ADLs) and related abilities are the most studied outcome among the included studies (n = 9). Occupational performance was assessed in three studies by using different instruments such as *the Child Occupational Self-Assessment *(*COSA*) (Meister & Salls, 2015), *the Canadian Occupational Performance Measure *(*COPM*) (Gentry et al., 2010) or video analysis (Campbell et al., 2015). In nine studies, specific ADL such as shopping (Lamash & Josman, 2021), mobility in the community (crossing street) (Josman et al., 2008), mealtime (Lo et al., 2009), toothbrushing (Lo et al., 2009) and play skills (Henning et al., 2016), were assessed with *the Test of grocery shopping skills *(*TOGSS*)*, the Pedestrian safety scale *(*PSS*)*,* duration of meals and % of non-feeding behaviors through video recording, the number of brushing strokes through video recording and plaque-revealing dye*,* and *the Test of Playfulness *(*ToP*)*,* respectively (Table 2)*.*

Social skills are the second most frequent study outcome among the included studies (n = 6). Two studies (Chen et al., 2015; Lee et al., 2016) assessed emotion recognition with non-standardized assessments such as video recording observation and questions regarding video-stories. Among the rest of the studies investigating social skills, several different measurement instruments were used. One study (Henning et al., 2016) used the *Conners' Comprehensive Behaviour Rating Scales-Parent *(*CCBRS-P*) to assess social and behavioral problems. One study (Gal et al., 2016) investigated social interaction assessing it with the *Friendship Observation Scale *(*FOS*)*.* One study (Hochhauser et al., 2018) measured conflict management and negotiation attitude using *the Five Factor Negotiation Scale *(*FFNS*) and *The ConflictTalk questionnaire*. Another (Parsons et al., 2019), assessed social imitation with *the Communication and Symbolic Behavior Scales *(*CSBS*) (Table 2).

Cognitive skills are the third most frequent study outcome among the included studies (n = 5). General cognitive functioning was assessed in two studies by the use of WebNeuro software (Lamash & Josman, 2021) and *the Mullen Scales of Early Learning *(*MSEL*) (Parsons et al., 2019). Specific cognitive aspects such as sustained attention (Cosper et al., 2009), time management (Janeslätt et al., 2014) and self-determination (Hatfield et al., 2017) were assessed with the *Gordon Diagnostic Systems, the Kit for assessing Time-processing ability in children *(*KaTid-Child*)*,* and *the AIR Self-Determination Scale *(*AIR*)*,* respectively (Table 2)*.*

Motor skills were assessed less frequently than cognitive skills in the included studies (n = 3). Among the motor skills assessed, we found: motor control (Cosper et al., 2009; Palsbo & Hood-Szivek, 2012; Wuang et al., 2010) assessed with *the Bruininks-Oseretsky Test of Motor Proficiency-Short Form* (Cosper et al., 2009), *the Beery-Buktenica Developmental Test of Visual-Motor Integration *(*VMI*) (Palsbo & Hood-Szivek, 2012), and *the Bruininks-Oseretsky Test of Motor Proficiency *(*BOTMP*) (Wuang et al., 2010); motor coordination assessed with the *Bruininks-Oseretsky Test of Motor Proficiency-Short Form* (Cosper et al., 2009), and handwriting, assessed with the Test of *Handwriting Skills-Revised *(*THS-R*) and *the Evaluation Tool of Children’s Handwriting *(*ETCH*) (Palsbo & Hood-Szivek, 2012) (Table 2).

Other outcomes and questionnaires were studied to a lesser extent in the included studies. Sensory integration was assessed (Wuang et al., 2010) with *the Test of Sensory Integration Function *(*TSIF*)*;* visual-spatial skills were assessed with *the Developmental test of visual-perceptual skills-2 *(*DTVP-2*) (Lee et al., 2013), speech generation was assessed using the Proloquo2Go trial score (Lorah et al., 2014), and intervention effectiveness was assessed using *the Goal Attainment Scaling *(*GAS*) (Ikuta et al., 2016) (Table 2).

### NT Used in OT Interventions in Children and Adolescents with ASD

NT used in the OT interventions in the included studies can be categorized into three clear types: OT interventions using computers (n = 12), OT interventions using iPad™ (n = 3) and OT interventions using another NT (n = 5) such as Personal Digital Assistant (PDA) (n = 2), assistive robot (n = 1), simulated Developmental Horse-Riding Program (SDHRP) (n = 1) and earmuffs and Noise-cancelling Headphones (n = 1).

### OT Interventions Using NT in Children and Adolescents with ASD

The specific characteristics of these interventions are summarized in Table 3. All interventions described in the included studies were exclusively carried out by occupational therapists (n = 20) (Campbell et al., 2015; Chen et al., 2015; Cosper et al., 2009; Gal et al., 2016; Gentry et al., 2010; Hatfield et al., 2017; Henning et al., 2016; Hochhauser et al., 2018; Ikuta et al., 2016; Janeslätt et al., 2014; Josman et al., 2008; Lamash & Josman, 2021; Lee et al., 2013, 2016; Lo et al., 2009; Lorah et al., 2014; Meister & Salls, 2015; Palsbo & Hood-Szivek, 2012; Parsons et al., 2019; Wuang et al., 2010), psychologists, teachers and research professors formed part of the intervention team in only three of them (Janeslätt et al., 2014; Palsbo & Hood-Szivek, 2012; Parsons et al., 2019). Computers were the most used NT in the included studies (n = 12). In general, intervention programs lasted between 1 and 15 weeks, although in two studies the intervention lasted 40 (Wuang et al., 2010) and 48 weeks (Hatfield et al., 2017). The intervention programs consisted of between 1 and 5 weekly sessions, but in one study there were 9 weekly sessions (Henning et al., 2016). Only two interventions carried out daily sessions (Lorah et al., 2014; Parsons et al., 2019). In general, the sessions lasted 45–60 min, except for one study (Janeslätt et al., 2014), which lasted 120 min. Some studies, did not state the duration of the intervention programs (Janeslätt et al., 2014; Josman et al., 2008; Lorah et al., 2014) or that of sessions performed (Ikuta et al., 2016).

### OT Interventions Using Computers

The most frequently used device in OT interventions is the computer (n = 12) (Campbell et al., 2015; Chen et al., 2015; Cosper et al., 2009; Gal et al., 2016; Hatfield et al., 2017; Henning et al., 2016; Hochhauser et al., 2018; Josman et al., 2008; Lamash & Josman, 2021; Lee et al., 2013, 2016; Lo et al., 2009). It is a highly versatile device with different intervention options that can be differentiated as follows:

#### Computer Applications

Two studies (Lee et al., 2013; Lo et al., 2009) used computer applications, aimed at training basic ADLs and improving visuo-perceptual skills. In both articles the intervention was performed exclusively by an occupational therapist. The duration of the intervention varied from 1 to 6 weeks with two to three sessions per week.

#### Virtual Environments

Two studies (Josman et al., 2008; Lamash & Josman, 2021) conducted an intervention using computer-based virtual environments to improve instrumental ADLs and social conversations. In both articles the intervention was performed exclusively by an occupational therapist. The duration of the intervention varied from 1 to 8 weeks, with a weekly session of 45 min.

#### Video Modeling

Four studies (Campbell et al., 2015; Gal et al., 2016; Hochhauser et al., 2018; Lee et al., 2016) conducted a video modeling intervention focused on emotion recognition, conflict resolution, social interaction and ADLs. All the interventions were performed exclusively by an occupational therapist. The duration of the intervention varied from 4 to 8 weeks with a 1-h weekly session.

#### Online Programs

One study (Hatfield et al., 2017) carried out an online program to prepare children for the transition to high school. The intervention was performed exclusively by an occupational therapist and lasted 12 months.

#### Augmented Reality

One study (Chen et al., 2015) used an augmented reality system to train social skills. The intervention was performed exclusively by an occupational therapist. The duration of the intervention was one and a half months with a total of seven sessions.

#### Non-invasive PC-Based Technology

One study (Cosper et al., 2009), used non-invasive pc-based technology to improve motor coordination and sustained attention. The intervention was performed exclusively by an occupational therapist. The duration of the intervention was 15 weeks with a 1-h weekly session.

#### Multiuser Tabletops Technology

One study (Henning et al., 2016) used a multi-touch collaboration computer interface table to improve collaboration skills. The intervention was performed exclusively by an occupational therapist. The duration of the intervention was 3 weeks with three to four forty-five-minute weekly sessions.

### OT Interventions Using iPads™

The second most used device was the iPad™ (n = 3) (Lorah et al., 2014; Meister & Salls, 2015; Parsons et al., 2019). This device was used to improve social, visuo-motor, imitation, language and ADL skills. The interventions were performed exclusively by an occupational therapist, except in one study (Parsons et al., 2019), in which a psychologist collaborated. The duration of the intervention varied from to 6 weeks to 3 months with two 25 min weekly sessions (Meister & Salls, 2015) or daily sessions (Lorah et al., 2014; Parsons et al., 2019).

### Interventions Using Another NT

Personal digital assistant (PDA): Two studies (Gentry et al., 2010; Janeslätt et al., 2014) used a PDA to train time management in daily life. One intervention was performed by an occupational therapist and the other by an occupational therapist and a special educator. In one study the duration of the intervention program was 40 days (Gentry et al., 2010) and in the other the duration was not stated (Janeslätt et al., 2014). Three ninety-minute sessions (Gentry et al., 2010) and an undisclosed number of 2-h sessions (Janeslätt et al., 2014) were performed.

#### Assistive Robot

One study (Palsbo & Hood-Szivek, 2012) used an assistive robot with haptic interface software to improve handwriting. The intervention was performed by an occupational therapist and a research professor. The duration of the intervention was 4 to 6 weeks with 4 weekly sessions.

#### Simulated Developmental Horse-Riding Program (SDHRP)

One study (Wuang et al., 2010) used a simulated developmental horse-riding program using JOBA® which focused on the development of motor skills and sensory integration. The intervention was performed exclusively by an occupational therapist. The duration of the intervention program was two twenty-week phases with 2 weekly sessions.

#### Earmuffs and Noise-Cancelling Headphones

One study (Ikuta et al., 2016) used earmuffs and noise-cancelling headphones to reduce behaviors related to auditory hyperreactivity. The intervention was performed by an occupational therapist. The intervention lasted for 6  weeks.

### Main Results of Included Studies

The specific results of each study are shown in Table 3. The effect of the interventions was positive in most of the articles, expressed as significant differences in the results either pre-post intervention or between the intervention group and control group. Seven studies (Campbell et al., 2015; Gentry et al., 2010; Janeslätt et al., 2014; Josman et al., 2008; Lamash & Josman, 2021; Lo et al., 2009; Meister & Salls, 2015) showed significant improvements in the performance of basic and instrumental ADLs, such as eating, personal hygiene, shopping, mobility in the community and time management in these activities. Five studies (Chen et al., 2015; Gal et al., 2016; Henning et al., 2016; Hochhauser et al., 2018; Lee et al., 2016) showed significant improvements in social skills, including collaboration, emotion recognition and conflict resolution. Two studies (Palsbo & Hood-Szivek, 2012; Wuang et al., 2010) reported significant improvements in motor skills, such as graphomotor skills and balance, and in sensory integration skills. One study (Lorah et al., 2014) showed significant improvements in communication and one study (Lee et al., 2013) found significant improvements in visual-spatial skills.

However, in some articles no significant results were reported. In two articles (Hatfield et al., 2017; Parsons et al., 2019), no significant results were reported when the groups were compared. In addition, one article (Cosper et al., 2009) only showed significant improvements in motor control, but not in the other study outcomes, sustained attention and motor coordination. In one study (Ikuta et al., 2016), of the two tools used in the intervention, only the results obtained by earmuffs were significant.

### Main Limitations of Included Studies

In Table 4 we show the main limitations reported by the authors of included studies, as well as other ethical aspects that could influence the interpretations of the obtained results, such as conflict of interest and funding sources.

The main limitation in included articles is small sample size, which was reported in twelve of them (Campbell et al., 2015; Gal et al., 2016; Gentry et al., 2010; Hatfield et al., 2017; Henning et al., 2016; Ikuta et al., 2016; Janeslätt et al., 2014; Josman et al., 2008; Lamash & Josman, 2021; Lee et al., 2013, 2016; Palsbo & Hood-Szivek, 2012). In seven studies, authors reported the absence of a control group (Cosper et al., 2009; Gentry et al., 2010; Henning et al., 2016; Ikuta et al., 2016; Janeslätt et al., 2014; Meister & Salls, 2015; Wuang et al., 2010), in six studies, authors reported the lack of monitoring and/or evaluation of the transference of results to the natural context was reported (Hatfield et al., 2017; Hochhauser et al., 2018; Ikuta et al., 2016; Lamash & Josman, 2021; Lorah et al., 2014; Meister & Salls, 2015), in four studies, authors reported a limited duration of the intervention (Campbell et al., 2015; Janeslätt et al., 2014; Lee et al., 2016; Meister & Salls, 2015), in three studies, authors reported a non-randomized and non-representative sample (Gal et al., 2016; Gentry et al., 2010; Hatfield et al., 2017), in three studies, authors reported a high withdrawal rate (Hatfield et al., 2017; Janeslätt et al., 2014; Lamash & Josman, 2021), in one study the dose and fidelity of treatment was lower than prescribed (Parsons et al., 2019), and in another, 43% of the sample refused to use any of the interventions (Ikuta et al., 2016). Finally, with regard to measurement instruments, one article highlights the absence of a valid instrument (Palsbo & Hood-Szivek, 2012), one used *KaTid-Child* and *Time Parent* scales, which have not previously been used to assess long-term effects (Janeslätt et al., 2014), and one used the *Five Factory Negotiation Scale*, which has a low internal consistency (Hochhauser et al., 2018).

Regarding declarations of interest, in seven articles (Campbell et al., 2015; Henning et al., 2016; Ikuta et al., 2016; Janeslätt et al., 2014; Lamash & Josman, 2021; Lee et al., 2016; Parsons et al., 2019) authors declared that they had no conflict of interest. In one article (Hatfield et al., 2017), the authors declare a conflict of interest, based on the fact that the first author of the manuscript that described the efficacy of the BOOST-A program is also the developer of the program. The remaining articles did not include any information regarding conflicts of interest.

Funding sources for the articles were mostly public. Six articles (Gentry et al., 2010; Hatfield et al., 2017; Hochhauser et al., 2018; Ikuta et al., 2016; Lee et al., 2016; Palsbo & Hood-Szivek, 2012) obtained funding through grants, scholarships and support from public institutions. In one article (Campbell et al., 2015), authors stated that they had not received any financial support. The remaining articles did not provide information on funding sources.

## Discussion

The present scoping review describes the characteristics of OT interventions using NT in the rehabilitation of children and/or adolescents with ASD. We reviewed the available knowledge in different databases and OT journals and identified 20 articles which investigated OT interventions using NT in children and adolescents with ASD. In the interventions described in these studies, researchers used 12 different technologies, of which the computer and iPad™ were most used. The main goal of the interventions was to increase the performance of basic and instrumental ADLs, social skills, cognitive skills, motor skills and, to a lesser extent, other abilities such as sensory integration or intervention effectiveness.

All included articles were published during the last 13 years. The fact that all of the articles were quite recent can be partly explained by the fact that the use of NT as a therapeutic tool is relatively new. In 1993 and 1997, the first medical entities for the evaluation of NT in healthcare were created, and were dedicated to examining the clinical, social, economic and legal consequences of the use of technologies (Vidal-España et al., 2007). As a result, research using NT in the health context could not begin until some years later, and even more so in those areas that have not been recognized as health professions, which is the case of OT in many countries. In fact, it is in the last 10 years that there have been published articles supporting the growth of technology in ASD interventions (Pandina, 2021), which has led to an increasing use of NT as a rehabilitation tool. To some extent, this could also be due to the current increase in the popularity of NT, the greater availability of technologies and the rapid technological development worldwide.

Among the included studies, those carried out in Asian countries (n = 9) and in the United States (n = 7) stand out. This could be explained by the fact that these countries have the highest prevalence of child-adolescent ASD in the world [World Health Organization (WHO), 2021], which makes ASD a relevant public health problem and, therefore a research priority. In contrast, the low representation of European countries, such as Spain, is noteworthy. This could be attributed, among other reasons, to the low prevalence of ASD in these countries (Málaga et al., 2019). In addition, recent studies have underlined the fact that Spain has a high level of obsolescence in terms of currently installed technological healthcare equipment [Federación Española de Empressas de Tecnología Sanitaria (Fenin) 2019] which makes research in this field difficult.

### NT Used in OT Interventions in Children and Adolescents with ASD

The most commonly used device in the interventions described in the included articles was the computer, followed by the iPad™ and, other technologies such as assistive robots, PDA, earmuffs and headphones and simulated developmental horse-riding programs. One clear reason for this is that computers are the most widely owned device in the population, and are present in most ASD children’s homes and schools (Ramdoss et al., 2011). The National Statistics Institute (INE) indicates the presence of computers in 81.4% of households (Instituto Nacional de Estadística, 2021). This popularity has made computers an easy device to implement in rehabilitation centers because children and adolescents with ASD are familiar with their use. In fact, previous studies indicated a clear preference of children with ASD for computers and their screens (Moore & Calvert, 2000).

In contrast, emerging NT such as robotics or augmented reality systems have been less used in the OT interventions described in included studies. This could be partly explained by the fact that OT interventions are mainly focused on the recovery of ADL in children and adolescents with ASD, and the evidence with regard to the use of emerging NT (i.e. robotics) in these children is specially related to social skills in communication (Kumazaki et al., 2020). In fact, the results of the interventions using robots showed that children with ASD achieve a higher level of task engagement when they interact with robots than with humans (Schadenberg et al., 2020). There has been too little research into the adequate necessary conditions of the interventions using emerging NT to generalize these results to human interactions (Pop et al., 2013).

### OT Interventions Using NT in Children and Adolescents with ASD

Most of the interventions described in included articles have been carried out exclusively by occupational therapists, without the support of a multidisciplinary team. We are surprised that other professionals collaborated in the interventions in only three studies (Janeslätt et al., 2014; Lorah et al., 2014; Parsons et al., 2019). Most of the OT interventions using NT were mainly focused on increasing ADLs, followed by social, cognitive and motor skills. This was as expected because we searched specifically for OT interventions, and these professionals are responsible for the rehabilitation of ADLs thorough occupation (Kuhaneck et al., 2020). Similarly, the fact that ASD children present several social difficulties, such as persistent impairments in social communication and social interaction in different contexts justifies the researchers' interest in social skills as a study outcome in the included studies [American Psychiatric Association (APA), 2002].

Surprisingly, no article has studied participation, which is a characteristic concept related to OT. A reason for this can be that occupation is closely related to participation. In fact, some authors point out that occupation is the “means” to rehabilitation and participation is the “end” (Larsson-Lund & Nyman, 2017) and this is could lead to confusion between the two terms and to use them indistinctly. Participation is included as a key aspect in a number of practice models in OT and is defined in the International Classification of Functioning, Disability and Health (ICF) as “involvement in a life situation” (WHO, 2001). Children and adolescents with ASD are particularly at risk of presenting restrictions in their participation. Previous studies have shown that children and adolescents with ASD participate in activities less frequently and with less variety than their peers with other disabilities or typical development (Rodger & Umaibalan, 2011; Shattuck et al., 2011). In this sense, the digitalization of society is changing the way OT is carried out as well as people's participation (Larsson-Lund & Nyman, 2019). NT can help occupational therapists to achieve their intervention goals, thanks to the simulation of real contexts and ADLs, in a personalized, interactive and novel way; but in a safe and familiar environment for children and adolescents with ASD, which facilitates and promotes their learning (Valencia et al., 2019) and therefore, in some way, also their participation.

The characteristics of the OT interventions using NT varied greatly among included studies in terms of intervention program duration, number of sessions, duration of the sessions and technology used. It is possible that this was an effect of the inclusion of different experimental studies in our scoping review, because often a case report or exploratory studies have a shorter intervention program duration than controlled trials. The duration of the sessions is the most stable parameter among included studies, and was usually between 45 and 60 min, which is a typical session length.

OT interventions using NT described in included studies have shown positive results in different aspects of children and adolescents with ASD, such as ADLs and social skills. These results could be conditioned by the limitations reported in the included studies, such as the small sample size, the absence of a control group, the limited duration of the interventions, the lack of follow-up, the lack of evaluation of the transference of the results to the natural context, and the selection of non-randomized and non-representative samples. However, these positive effects on ADLs and social skills have also appeared in previous published reviews (Becker et al., 2016; Vidal-España et al., 2007). Although not in the OT field, the reviews by Wainer et al. (Wainer & Ingersoll, 2011) and Tanner et al. (Tanner et al., 2015), described the use of NT as adjuvants to conventional intervention to improve facial and emotion recognition, social problem solving and social behaviors in children with ASD. Therefore, NT could be seen as a useful and complementary tool to OT intervention in children and/or adolescents with ASD.

### Practical and Clinical Implications

The interventions presented in this review are part of an emerging area of research, and offer occupational therapists a great opportunity for research and clinical practice. This review provides information on outcomes, types of interventions and technologies without focusing on a specific area or variable of intervention, providing a broader view of the use of NT in OT interventions in children and adolescents with ASD. Professionals could use the results to develop intervention programs based on NT, and include the different tools presented in their intervention sessions with children and adolescents with ASD. We also provide a summary of current evidence that can support the performance of evidence-based OT practice. However, occupational therapists should consider the results of this scoping review carefully before determining and developing intervention plans for their patients.

### Strengths and Limitations of this Scoping Review

Our scoping review presents some limitations that should be considered when interpreting its results. Although we performed a systematic peer review to ensure scientific rigor, the lack of information reported in included studies, the publication bias which limits null results of the interventions, and the selection bias are limitations for the majority of reviews. Regarding inclusion criteria, we only included articles published in English and/or Spanish and with full text available, so we may have excluded studies that could provide relevant information published in another language. Regarding the search strategy, the terms that define ASD and NT were difficult to define. In recent years ASD classification has been modified and there is no consensus on the terminology used in existing articles. Therefore, we have used the terms included in the DSM-IV [American Psychiatric Association (APA), 2002], which categorizes ASD as: Autistic Disorder, Pervasive Developmental Disorders, Rett Syndrome, Childhood Disintegrative Disorder and Asperger Syndrome. As a result, studies using other terms such as hyperactive disorder associated with mental retardation and stereotyped movements present in the ICD-10 may have been overlooked. Something similar occurred with the included terms in NT. We decided to include different terms that had already been used in previous published reviews (Sandgreen et al., 2020; Strubbia et al., 2020). Moreover, in this review we only included those articles in which occupational therapists were one of the professionals who performed the interventions in ASD. Thus, it is possible that we overlooked articles in which occupational therapists were involved in the intervention, but this was not clearly specified in the study, which favored the selection bias. With regard to included studies, we need to point out that we have only included experimental studies which could contain biases related to this type of study design. In addition, in some of them, measurement instruments which were not validated or standardized were used (Campbell et al., 2015; Chen et al., 2015; Josman et al., 2008; Lee et al., 2013, 2016; Lo et al., 2009; Lorah et al., 2014). Moreover, we have not performed an assessment of the quality of the included studies, which means that some of the articles included in this review may have a low methodological quality. However, we have presented the main reported limitation of every included study in Table 3 and other aspects related to the quality of the results, such as conflicts of interest in Table 4. Finally, the high level of variability in terms of assessment tools, outcomes, sample size and duration of intervention among included articles makes it difficult to compare included studies and to draw conclusions. Thus, the results of this scoping review must be interpreted with caution.

Our scoping review also presents some strengths. We should highlight that we have not found other reviews in the scientific literature aimed at describing OT interventions using NT in children and adolescents with ASD. Thus, our scoping review may provide relevant information in a field that has been investigated very little. In addition, this review provides information regarding current gaps in knowledge from which to begin future lines of research: (i) OT interventions using NT in children and adolescents with ASD have been investigated very little in European countries; (ii) There has been very limited research into OT interventions using smartphones and emerging technologies such as robotics; (iii) Multidisciplinary intervention in children and adolescents with ASD using NT is scarce; (iv) OT interventions using NT in children and adolescents with ASD varied greatly in duration and number of sessions. Moreover, we underline the necessity of performing further studies with a larger sample size, containing a control group, with a representative and random sample, and in which validated assessment tools are used.

## Conclusion

The computer was the most frequently used NT in OT interventions in children and adolescents with ASD, followed by iPad™ and other technologies such as augmented reality or robots. The interventions were mainly carried out exclusively by an occupational therapist and were focused on the improvement of ADLs and social skills. The duration of the interventions lasted between 1 week and 12 months, and the number of sessions ranged between 1 daily session to 5 per week. However, the characteristics of the OT interventions using NT in children and adolescents with ASD varied greatly between studies, so further studies are needed to help quantify and define these parameters.

## Supplementary Information

Below is the link to the electronic supplementary material.

Supplementary file1 (PDF 549 KB)
